# Population Pharmacokinetics of Ropeginterferon Alfa-2b: A Comparison Between Healthy Caucasian and Chinese Subjects

**DOI:** 10.3389/fphar.2021.673492

**Published:** 2021-05-28

**Authors:** Min Zhu, Mei-xia Wang, Zi-ran Li, Wei Wang, Xia Su, Zheng Jiao

**Affiliations:** ^1^ Department of Pharmacy, Shanghai Chest Hospital, Shanghai Jiao Tong University, Shanghai, China; ^2^ School of Basic Medicine and Clinical Pharmacy, China Pharmaceutical University, Nanjing, China; ^3^ National Clinical Trial Institution Office and Phase I Study Ward, Beijing Youan Hospital, Capital Medical University, Beijing, China; ^4^ Department of Pharmacy, Huashan Hospital, Fudan University, Shanghai, China; ^5^ PharmaEssentia Biotechnology (Beijing) Limited, Beijing, China; ^6^ Pharmacometrics, Earnest and Ye-sayer, Beijing, China

**Keywords:** ropeginterferon alfa-2b, population pharmacokinetics, Chinese, Caucasian, nonlinear mixed effect modeling, ethnic difference, target-mediated drug disposition

## Abstract

**Objective:** To develop a population pharmacokinetic (PK) model for ropeginterferon alfa-2b and to compare its PK properties between Caucasian and Chinese populations.

**Methods:** A population PK model was developed based on data from two phase I clinical trials conducted in Caucasian and Chinese individuals, to evaluate the influence of ethnicity on the PKs of ropeginterferon alfa-2b.

**Results:** We included 456 observations from 30 healthy Caucasian subjects and 438 observations from 27 healthy Chinese subjects in the population PK analysis. The PKs of ropeginterferon alfa-2b were best described by a one-compartment quasi-equilibrium approximated target-mediated drug disposition model with first-order absorption and absorption lag times. The typical value (relative standard error%) of apparent clearance (*CL/F*) and volume of distribution of ropeginterferon alfa-2b in 70-kg subjects were 0.778 (12%) L/day and 2.32 (14%) L, respectively. Body weight was the only significant factor affecting the *CL/F*. There were no obvious differences in the PK properties of ropeginterferon alfa-2b, and predicted steady-state exposure was similar in the Chinese and Caucasian populations.

**Conclusion:** No significant ethnic differences in ropeginterferon alfa-2b PKs were observed between the Chinese and Caucasian populations.

## Introduction

Interferon (IFN) is produced in influenza virus-infected chick embryo cells, which has the ability to interfere with viral replication and induce resistance to viral infection. It has been widely used in the treatment of chronic hepatitis B (Janssen et al., 2005), hepatitis C (Rivero-Juarez et al., 2014), acquired immunodeficiency syndrome (Husak et al., 1997), and cancer (Bottomley et al., 2009). There are three principal routes of IFN elimination: through the liver, the kidneys, or via interactions with IFN receptors (Bocci, 1987). Standard IFNs have short serum half-lives (approximately 6 h) (Wills, 1990), which limits their use. Polyethylene glycol-modified (PEGylated) forms of IFN have considerably long half-lives and provide a means of improving patient compliance (Pedder, 2003). The first-generation long-acting IFN PEG-Intron^®^ (Schering Corporation, 2001) from Merck and the second-generation Pegasys^®^ (Genentech Inc., 2002) from Roche have been approved by the United States Food and Drug Administration and European Medicines Agency (EMA), to treat chronic hepatitis B and hepatitis C. Both drugs are administered weekly via subcutaneous (SC) injection.

Ropeginterferon alfa-2b (BESREMi^®^) is a third-generation long-acting IFN injection marketed in the European Union for the treatment of polycythemia vera (Gisslinger et al., 2015). Ropeginterferon alfa-2b contains a PEGylated isomer at only one position (Illes et al., 2020), whereas PEG-Intron^®^ and Pegasys^®^ have 13 and six positional isomers, respectively (Foster, 2004). The single isomer entity of ropeginterferon alfa-2b and the monopegylation at the proline chain of the N-terminal of the moiety can reduce the variabilities caused by different isomers (Foser et al., 2003). The usual starting dose is 100 μg every 2 weeks (Q2W), which can be gradually increased to a maximum dose of 500 μg Q2W until a sufficiently low and stable red blood cell count is achieved (EMA, 2021). Compared to the weekly dose regimen of PEG-Intron^®^ and Pegasys^®^, the 2-weeks dosing interval makes ropeginterferon alfa-2b more convenient in clinical use.

A non-compartment PK analysis (NCA) of ropeginterferon alfa-2b has been conducted, using PK data from a phase I clinical trial conducted in healthy Caucasian volunteers. The dose proportionality analysis suggested more than proportional kinetics with an increasing dose, indicating that ropeginterferon alfa-2b might have a nonlinear PK (EMA, 2019). Moreover, although we have information for PK, efficacy, and safety of ropeginterferon alfa-2b in Caucasians, little was known in Chinese populations.

Here, intense sampling PK data pooled from two phase I studies conducted in Caucasians and Chinese were included to develop a population PK model of ropeginterferon alfa-2b for the first time. The population PK analysis aimed to 1) describe and explain the nonlinear PKs of ropeginterferon alfa-2b and 2) investigate the potential PK ethnic differences between Caucasian and Chinese populations.

## Methods

### Study Design

PK data from two phase I clinical trials of ropeginterferon alfa-2b (BESREMi^®^, PharmaEssentia Corp., Taipei) conducted on healthy Caucasian subjects (Study A09–102) and healthy Chinese subjects (Study A17–101), were pooled into analysis.

Study A09–102 was a single center, randomized double-blind, active-control, single dose escalation study conducted by Anapharm Inc. (Montréal, QC, Canada). In total, 48 adult male Caucasian subjects aged 18–45 years were randomized into six cohorts, with each evaluating one dose. Within each dosing cohort, two subjects received a single SC dose of Pegasys^®^ (180 μg) and the other six received a single SC injection of ropeginterferon alfa-2b (24, 48, 90, 180, 225, or 270 μg). Blood samples were collected pre-dosing and 1, 3, 6, 9, 12, 16, 24, 36, 48, 72, 96, 120, 144, 168, 192, 240, 288, 336, 504, and 672 h afterward.

Study A17–101 was a single center, randomized open-label, active-control, single dose escalation study conducted at the Beijing Youan Hospital (Beijing, China). Forty healthy male or female Chinese subjects aged 18–45 years were randomly assigned to four cohorts as follows: three ropeginterferon alfa-2b cohorts (*n* = 10 each) that received a single SC dose of 90, 180, or 270 μg ropeginterferon alfa-2b, and a Pegasys^®^ cohort that received a single SC dose of 180 μg Pegasys^®^ (*n* = 10). The proportion of males or females was no less than one-third in each cohort. Blood samples were collected pre-dosing and 1, 3, 6, 9, 12, 16, 24, 36, 48, 72, 96, 120, 144, 192, 240, 336, 504, and 672 h afterward.

The two studies were intended to investigate the PKs of ropeginterferon alfa-2b. Pegasys^®^ was chosen as the comparator in both studies since it is the typical drug of the 40-kDa long-acting PEGylated IFNs and expected to have comparable PKs with ropeginterferon alfa-2b.

Written informed consent was obained from all study subjects. Clinical trial A09–102 was reviewed and approved by the Quebec Institutional Review Board, Canada. Clinical trial A17–101 was reviewed and approved by the Ethics Committee of Beijing Youan Hospital affiliated to Capital Medical University, Beijing, China. Both studies were conducted in accordance with the International Council for Harmonization Good Clinical Practice guidelines and the Declaration of Helsinki 2004. Data from the subjects administered ropeginterferon alfa-2b were included in the PK analysis.

### Bioassays

Serum ropeginterferon alfa-2b concentrations were determined using the validated double antibody sandwich method by Pharmaron Biotechnology Co., Ltd. (Boston, MA, United States) in study A09–102, and by Xuzhou Jiasheng Pharmaceutical Technology Co., Ltd. (Xuzhou, China) in study A17–101. The bioassay method has been cross-validated in the two laboratories. The coefficient of variation of the bioassay was <10%, with a lowest limit of quantification of 50.0 pg/ml and a calibration range of 50.0–2,800 pg/ml.

### Non-Compartment Analysis

PK parameters were estimated through a NCA using the standard methods. The peak concentration (C_max_) was obtained directly from the concentration-time curve and the area under the concentration-time curve (AUC) was calculated using the linear trapezoidal rule. All calculations were performed using the R package PKNCA (version 0.9.4) (Denney et al., 2015).

### Population Pharmacokinetic Modeling

PK analysis was performed using the nonlinear mixed effects modeling software (NONMEM, version 7.3, ICON plc, Ellicott City, MD, United States) with a gFortran compiler (version 4.6.0, http://gcc.gnu.org/fortran/). Pre- and post-processing were conducted using Perl-speaks-NONMEM (version 4.9.0, https://uupharmacometrics.github.io/PsN/), Pirana (version 2.9.9, https://www.certara.com/software/pirana-modeling-workbench/), and R (version 3.6.1, http://www.r-project.org/). The first-order conditional estimation method, with *η-ε* interaction option, was used throughout model development.

#### Base Model

A first-order absorption with or without the lag time was investigated for modeling the SC absorption of ropeginterferon alfa-2b. A one-compartment model was selected according to the previous reports of PEG-Intron^®^ (Jen et al., 2001) and Pegasys^®^ (Saleh, 2017) as well as the diagnostic plot. Because nonlinear PKs were revealed by the dose proportionality analysis in Caucasians (EMA, 2019), an empirical Michaelis-Menten (MM) elimination model and a mechanism-based target-mediated drug disposition (TMDD) model were examined. Akaike information criterion (AIC) (Yamaoka et al., 1978) and Bayesian information criterion (BIC) (Dunne, 1993) were used to assess the candidate structural models.

**FIGURE 1:**
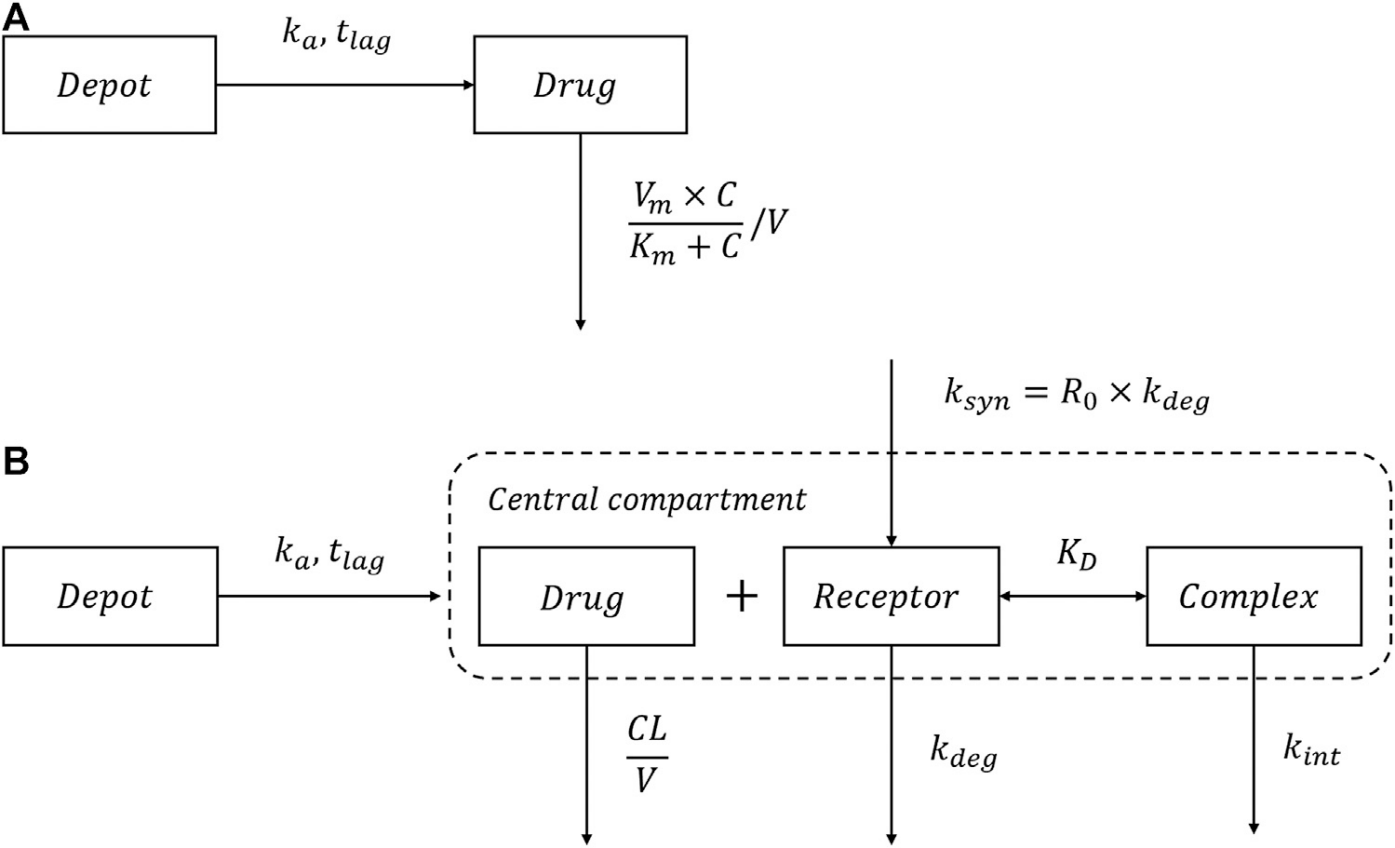
Schematic of the MM model and TMDD model. **(A)** The Michaelis-Menten (MM) elimination model; **(B)** The quasi-equilibrium approximated target-mediated drug disposition (TMDD) model. $k_{a}$: absorption rate; $t_{lag}$: absorption lag time; $V_{m}$: the maximum clearance; $K_{m}$: the MM constant; C: the drug concentration in the central compartment; CL: clearance; V: volume of distribution; $k_{syn}$: synthesis rate; $R_{0}$: receptor baseline concentration; $k_{deg}$: degradation rate; $K_{D}$: equilibrium dissociation constant; $k_{int}$: internalization rate.

The schematic of the MM model is shown in Figure 1A. The dose-dependent clearance can be described by Eq. 1:

$$\begin{array}{c} CL=\frac{V_{m}\cdot C}{K_{m}+C} \end{array}$$ (1)

where $V_{m}$ is the maximum clearance; $K_{m}$ is the MM constant; C is the drug concentration in the central compartment.

The schematic of the TMDD model is shown in Figure 1B. Because the concentrations of the receptor and drug-receptor complex were not available, the initial binding process for the general TMDD could not be sufficiently identified (Ma, 2012). Thus, the quasi-equilibrium (QE) approximated TMDD model (Mager and Krzyzanski, 2005) was employed, where the parameter $K_{D}$ (equilibrium constant) was used rather than $k_{on}$ (association rate) and $k_{off}$ (dissociation rate) to describe the receptor binding at quasi-equilibrium state.

As shown in Figure 1B, after SC administration, ropeginterferon alfa-2b enters the central compartment with first-order absorption rate $\left(k_{a}\right)$:

$$\begin{array}{c} \frac{\text{d}A_{depot}}{\text{d}t}=-k_{a}\cdot A_{depot}\; \end{array}$$ (2)

All processes, including receptor binding, receptor degradation, and drug-receptor complex internalization occur in the central compartment. Both drugs and receptors comprise two components: free drugs/receptors and complexes. Thus, the total drugs and receptors can be determined using Eqs 3, 4, respectively:

$$\begin{array}{c} \frac{\text{d}A_{total}}{\text{d}t}=k_{a}\cdot A_{depot}\;-\frac{CL}{V}\cdot A_{free}-k_{int}\cdot \left(A_{total}-A_{free}\right)\; \end{array}$$ (3)

$$\begin{array}{c} \frac{\text{d}R_{total}}{\text{d}t}=k_{syn}-k_{deg}\cdot R_{total}-\left(k_{int}-k_{deg}\right)\cdot \left(C_{total}-C_{free}\right)\; \end{array}$$ (4)

where $A_{total}$ and $A_{free}$ are the total and free drug amount in the central compartment, respectively; $C_{total}$ and $C_{free}$ are the total and free drug concentration, respectively; $R_{total}$ is the total receptor concentration;  V is the volume of distribution of the central compartment; $k_{syn}$ is the receptor synthesis rate; $k_{deg}$ is the receptor degradation rate; $k_{int}$ is the internalization rate of the drug-receptor complex. The initial condition for $R_{total}$ can be expressed as shown in Eq. 5:

$$\begin{array}{c} R_{total}\left(0\right)=R_{0}=\frac{k_{syn}}{k_{deg}} \end{array}$$ (5)

where $R_{0}$ is the baseline receptor concentration. The relationship between $K_{D}$ and $C_{free}$ can be described by Eq. 6:

$$\begin{array}{c} C_{free}=\frac{1}{2}\cdot \left[\left(C_{total}-R_{total}-K_{D}\right)+\sqrt{{\left(C_{total}-R_{total}-K_{D}\right)}^{2}+4\cdot K_{D}\cdot C_{total}}\right]\; \end{array}$$ (6)

The total drug amount (Eq. 3) can also be expressed in terms of free drug (Gibiansky et al., 2008):

$$\begin{array}{c} \frac{\text{d}A_{total}}{\text{d}t}=k_{a}\cdot A_{depot}\;-\frac{CL}{V}\cdot A_{free}-\frac{R_{total}\cdot k_{int}\cdot A_{free}}{K_{D}+C_{free}}\; \end{array}$$ (7)

Thus, the total systemic clearance of ropeginterferon alfa-2b can be calculated by Eq. 8:

$$\begin{array}{c} CL_{total}=CL_{linear}+CL_{nonlinear}=CL+\frac{R_{total}\cdot k_{int}\cdot V}{K_{D}}\cdot \left(1-\frac{C_{free}}{C_{free}+K_{D}}\right) \end{array}$$ (8)

where $CL_{linear}$ represents linear clearance which can be directly estimated by the model (CL) and $CL_{nonlinear}$ represents nonlinear clearance which is derived from a combination of target-mediated parameters ($R_{total}$, $K_{D}$ and $k_{int}$) (Gibiansky et al., 2008; Vu et al., 2017).

Between-subject variability (BSV) was estimated using an exponential function, as described in Eq. 9:

$$\begin{array}{c} p_{i,j}=p_{pop,j}\times exp\left(\eta_{i,j}\right) \end{array}$$ (9)

where $p_{i,j}$ is the value of parameter j for the ith individual; $p_{pop,j}$ is the typical value of parameter j; and $\eta_{i,j}$ is the BSV of the parameter, which follows a normal distribution with a mean of 0 and a variance of $\omega^{2}$.

Residual unexplained variability (RUV) was modeled using additive (Eq. 10), proportional (Eq. 11), and combined proportional and additive (Eq. 12) models as follows:

$$\begin{array}{c} Y=IPRED+\varepsilon_{add} \end{array}$$ (10)

$$\begin{array}{c} Y=IPRED\times \left(1+\varepsilon_{prop}\right) \end{array}$$ (11)

$$\begin{array}{c} Y=IPRED\times \left(1+\varepsilon_{prop}\right)+\varepsilon_{add} \end{array}$$ (12)

where Y is the observation; IPRED is the individual prediction; $\varepsilon_{prop}$ is the proportional residual variability, which follows a normal distribution with a mean of 0 and a variance of $\sigma_{prop}^{2}$, and $\varepsilon_{add\;}$ is the additive residual variability, which follows a normal distribution with a mean of 0 and a variance of $\sigma_{add}^{2}$.

#### Covariate Model

The covariates investigated in this study were subject demographics [age, sex, ethnicity, weight, body mass index (BMI), and body surface area (BSA)]. Because all subjects included in this study were healthy volunteers, the laboratory tests (alkaline phosphatase, alanine glutamate transferase, aspartate aminopeptidase transferase, total bilirubin, albumin, serum creatinine, hemoglobin level, hematocrit, white blood cell count, and platelet count) were within the normal ranges. The effects of these factors could not be identified, and they were not included in the covariate analysis.

First, we visually inspected the relationships between the post hoc Bayesian estimates and covariates, to identify potential covariates. These were then tested using a stepwise forward inclusion and a backward elimination procedure. In the forward inclusion process, covariates were considered significant if they decreased the objective function value (OFV) by > 3.84 ($\chi^{2}$ test, degree of freedom (df) = 1, *p* < 0.05). In the backward procedure, covariates that resulted in an OFV increase >6.63 ($\chi^{2}$ test, df = 1, *p* < 0.01) were retained in the model. Covariates were also evaluated based on their clinical and biological plausibility.

Continuous covariates were modeled using a power function (Eq. 13):

$$\begin{array}{c} p_{i}=p_{pop}\times {\left(\frac{cov_{i}}{cov_{pop}}\right)}^{\theta } \end{array}$$ (13)

For categorical covariates, such as sex, a proportional function was used, as described in Eq. 14:

$$\begin{array}{c} p_{i}=\{\begin{array}{cc} p_{pop} & \;if\;male\\ p_{pop}\times \theta & if\;female \end{array} \end{array}$$ (14)

where, in both the models, $p_{pop}$ is the typical value of the PK parameter and $p_{i}$ is the parameter value for the ith individual. For continuous covariates, $cov_{i}$ is the value of the covariates for the ith individual; $cov_{pop}$ is the median value for continuous covariates, and θ represents the influence index of covariates on the parameters.

#### Model Evaluation

The final model was evaluated using goodness-of-fit (GOF) plots, a visual predictive check (VPC) (Yano et al., 2001), and bootstrapping. A VPC with 1,000 simulations was performed. The median and 10th and 90th percentiles of the distributions of the simulated concentration values were calculated and assessed after overlaying the observations. A non-parametric bootstrap method with 1,000 iterations was used to assess the stability of the model. The 5th to 95th percentiles for bootstrap replicates were obtained and compared with the parameter estimates from the final model.

### Simulation

Monte Carlo simulations were conducted based on the final established model, to compare the PKs of ropeginterferon alfa-2b in the two populations (*n* = 1,000 each). After multiple SC doses of 100 or 200 μg Q2W, the area under the time-concentration curve at steady state (AUC_ss_) for Caucasian and Chinese populations was simulated using Simulx (version 2019R12, Lixoft SAS, Antony, France) (Lixoft, 2019).

## Results

### Subjects

Of the 66 subjects administered ropeginterferon alfa-2b, nine (six Caucasian subjects from the study A09–102 and three Chinese subjects from the study A17–101) were excluded from the PK analysis because of drop-out. Therefore, 57 subjects with 894 concentrations were finally included in the study. A total of 456 observations were from 30 Caucasian subjects, and 438 observations were from 27 Chinese subjects.

Table 1 summarizes the demographic characteristics of the subjects enrolled in the population PK analysis. The number and percentage for categorical variables and the mean and standard deviation for continuous variables were presented. The differences between Caucasians (Study A09–102) and Chinese (Study A17–101) were compared by χ^2^ test for categorical variables and *t* test for continuous variables. Subjects in study A09–102 were all males and study A17–101 had a sex ratio of 15: 12 (male: female). The male/female ratios in A17–101 were 5:4, 5:3, and 5:5 for 90, 180, and 270 μg cohorts, respectively. Ages in the two ethnic groups were comparable (32.3 ± 6.9 vs. 31.6 ± 6.19), whereas the body weight was 14% lower in the Chinese population than the Caucasian population (68.1 ± 10 vs. 79.4 ± 9.41 kg).

**TABLE 1:** Demographic characteristics of subjects.

	Total (*n* = 57)	Study A09–102 (*n* = 30)	Study A17–101 (*n* = 27)	*p* value
Ethnicity				<0.001
Caucasian	30 (52.63)	30 (100)	0 (0)	
Chinese	27 (47.37)	0 (0)	27 (100)	
Sex				<0.001
Male	45 (78.95)	30 (100)	15 (55.56)	
Female	12 (21.05)	0 (0)	12 (44.44)	
Age (years)	32.3 ± 6.52	32.3 ± 6.9	31.6 ± 6.19	0.657
Height (cm)	171 ± 9.32	176 ± 7.51	166 ± 8.51	<0.001
Weight (kg)	74 ± 11.2	79.4 ± 9.41	68.1 ± 10	<0.001
Body mass index (kg/m^2^)	25.2 ± 2.68	25.7 ± 2.43	24.7 ± 2.89	0.166
Body surface area (m^2^)	1.89 ± 0.182	1.98 ± 0.147	1.78 ± 0.163	<0.001
Alkaline phosphatase (U/L)	76.3 ± 20.7	73.1 ± 19.6	79.9 ± 21.6	0.223
Alanine glutamate transferase (U/L)	23.9 ± 13.6	26.4 ± 16.1	21.1 ± 9.77	0.147
Aspartate aminopeptidase transferase (U/L)	20.4 ± 5.26	20.5 ± 5.82	20.3 ± 4.68	0.865
Total bilirubin (μmol/L)	12.1 ± 4.98	10.9 ± 5.45	13.4 ± 4.09	0.053
Serum creatinine (mg/dl)	0.793 ± 0.137	0.834 ± 0.106	0.747 ± 0.154	0.014
Estimated glomerular filtration rate (ml/min·1.73 m^2^)	109 ± 22.1	99.4 ± 12	120 ± 15.8	<0.001
Hemoglobin (g/L)	148 ± 12.7	151 ± 8.87	145 ± 15.4	0.057
Hematocrit (%)	43.1 ± 3.36	43.6 ± 2.31	42.6 ± 4.22	0.288
White blood cell count (10^9^/L)	6.15 ± 1.38	5.92 ± 1.34	6.4 ± 1.41	0.191
Platelet count (10^9^/L)	239 ± 48.6	225 ± 47.4	254 ± 45.9	0.020

Values shown are the number (n) and percentage for categorical covariates and mean ± standard deviation for continuous covariates, respectively. *p* values were calculated by χ^2^ test for categorical covariates and *t* test for continuous covariates, respectively.

### Non-Compartment Analysis

**FIGURE 2:**
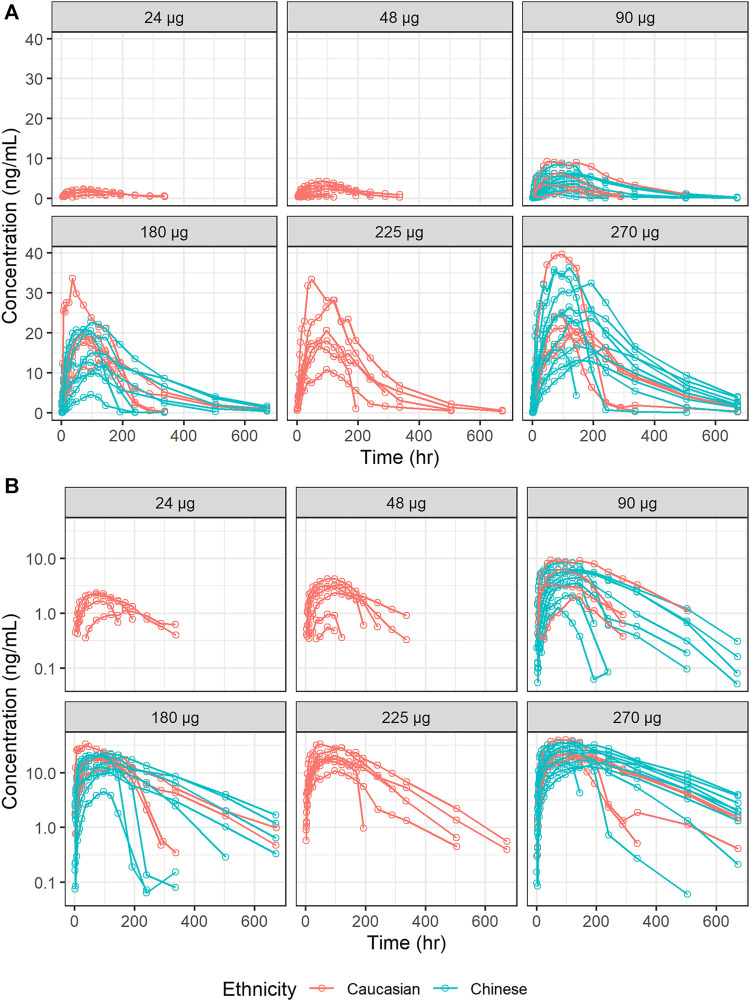
Concentration-time curves of ropeginterferon alfa-2b stratified by dose. **(A)** Ordinary coordinate; **(B)** Semi-logarithmic coordinate. Red points and lines represent Caucasian populations; blue points and lines represent Chinese populations.

**FIGURE 3:**
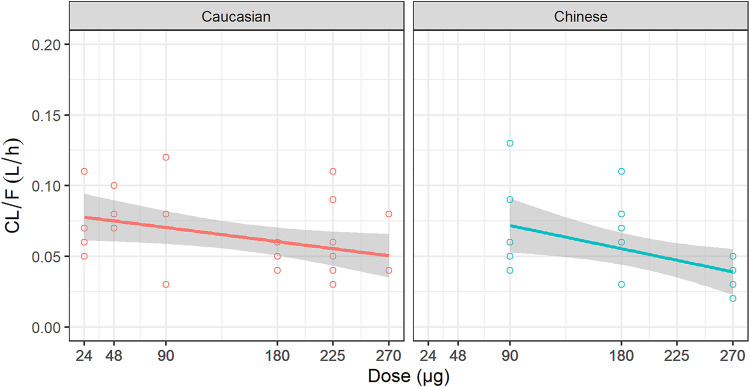
Relationship between dose and CL/F. *CL/F*: the apparent clearance derived from non-compartment analysis.

The estimates of PK parameters, including C_max_, AUC from time zero to the time of the last quantifiable concentration (AUC_0–t_), and AUC from time zero extrapolated to infinity (AUC_0–inf_), are shown in Supplementary Table S1. The concentration-time curves stratified by dose are shown in Figure 2, which implied that Caucasians and Chinese populations had similar PK profiles. *CL/F* derived from the NCA decreased with an increase in dose (Figure 3).

### Population Pharmacokinetic Modeling

#### Base Model

First-order absorption with a lag time ($t_{lag}$) better described the absorption phase than absorption without a $t_{lag}$. The QE approximated TMDD model had lower AIC (1387.9 vs. 1588.1) and BIC (1469.5 vs. 1640.9) values than the MM elimination model, thus was chosen as the structural model. In addition, the RUV was best described by the combined proportional and additive model.

The condition number of the TMDD model was too large (2.4 × 10^7^), indicating that the model was over-parameterized and needed to be simplified (William Bachman, 2003). The relative standard errors of the estimates of $K_{D}$ (56%) and $k_{int}$ (77%) were larger than 50%. Therefore, these parameters were fixed according to the initial estimation as a clue (Chaturvedula, 2016). The $K_{D}$ was fixed at 0.142 ng/ml, and $k_{int}$ was fixed at 0.0788 h^−1^. In addition, the shrinkage values for $R_{0}$ (30%) and $K_{D}$ (69%) were too high, indicating that the data did not support the BSV estimation for these parameters (Savic and Karlsson, 2009). Therefore, only BSVs for *CL/F*, *V/F*, and *k*_*a*_ were estimated.

The sensitivity analysis was performed for these two parameters ($K_{D}$ and $k_{int}$). The fixed parameter values were changed to 0.5, 0.75, 1.5, and two times their initial values to investigate whether these fixed settings had significant impacts on *CL/F*, *V/F*, and *k*_*a*_ (Supplementary Table S2). The estimates of these parameters varied by <20%, indicating that fixing the $K_{D}$ and $k_{int}$ values had insignificant effect on *CL/F*, *V/F*, and *k*_*a*_ estimation.

#### Covariate Model

Of all the covariates included in this study, ethnicity, sex, weight, BMI, and BSA showed correlations with *CL/F* and *k*_*a*_ by visual inspection. These were then included into the population PK model for a quantitative analysis. Among the three covariates which were highly correlated (namely, weight, BMI, and BSA), weight was selected.

The stepwise covariates selection is shown in Supplementary Table S2. Only weight was selected in forward inclusion as a significant factor affecting *CL/F*, as it resulted in an OFV decrease of 8.309 (*p* = 0.004). After backward elimination, weight was still retained in the final model as follows:

$$\begin{array}{c} CL/F=0.778{\left(\frac{WT}{70}\right)}^{0.927} \end{array}$$ (15)

No significant effect of ethnicity was found on all PK parameters, including *CL/F*, *V/F*, and *k*_*a*_. Parameter estimates for the final population PK model are presented in Table 2.

**TABLE 2:** Parameter estimates of the final model and bootstrap evaluation.

Parameters	Estimate (RSE%)	Shrinkage (%)	Bootstrap median (5–95%)
Typical value			
CL/F (L/day)	0.778 (12)	/	0.779 (0.653–0.905)
Impact of body weight	0.927 (43)	/	0.987 (0.347–1.556)
V/F (L)	2.32 (14)	/	2.259 (1.650–3.098)
k_a_ (1/day)	0.14 (14)	/	0.138 (0.117–0.166)
t_lag_ (h)	0.426 (9)	/	0.502 (0.045–0.822)
R_0_ (ng/ml)	0.111 (31)	/	0.126 (0.037–1.176)
k_int_ (h^−1^)	0.0788 (Fixed)	/	/
k_deg_ (h^−1^)	0.544 (44)	/	0.479 (0.046–2.167)
K_D_ (ng/ml)	0.142 (Fixed)	/	/
Between subject variability (CV%)			
CL/F	35.7 (14)	15	32.2 (23.2–42.3)
V/F	90.8 (15)	5	76.0 (61.8–96.3)
k_a_	63.5 (17)	12	56.6 (44.6–68.6)
Residual unexplained variability			
Proptional (%)	18.7 (2)	8	18.06 (14.7–24.2)
Additive (ng/ml)	0.342 (3)	8	0.331 (0.113–0.487)

*CL/F*, apparent clearance; *V/F*, apparent volume of distribution; *k*_*a*_, absorption rate; *t*_*lag*_, absorption lag time; *R*_*0*_, receptor baseline concentration; *k*_*int*_, internalization rate of drug-receptor complex; *k*_*deg*_, degradation rate of receptor; *K*_*D*_, equilibrium dissociation constant of drug-receptor binding; RSE, relative standard error; CV, coefficient of variation.

CV was calculated using $\sqrt{\exp\left(\omega^{2}\right)-1}\times 100$, where $\omega^{2}$ was the variance of the between subject variability.

#### Model Evaluation

**FIGURE 4:**
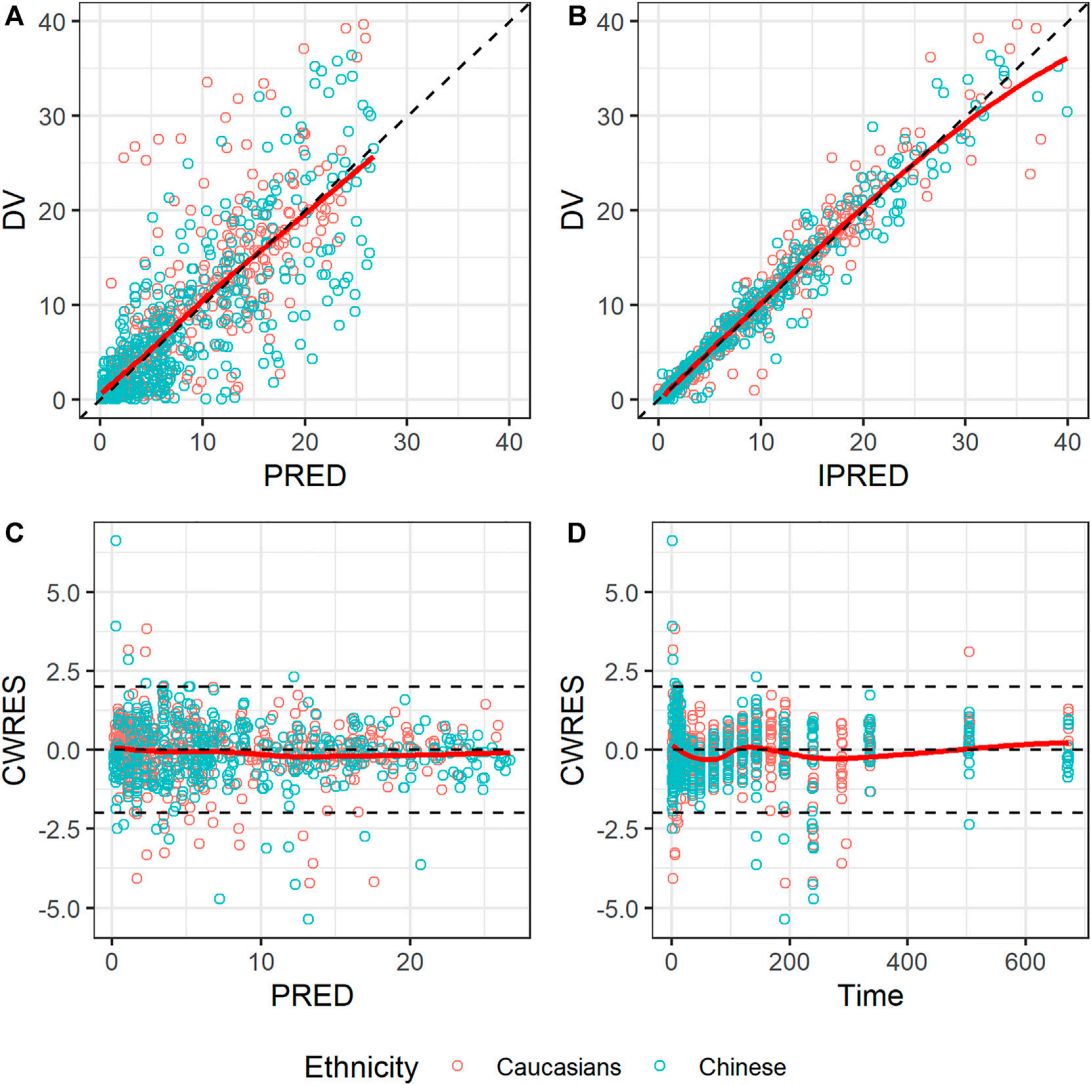
Goodness-of-fit plots for the final model. **(A)** Observed concentration (DV) vs. population prediction (PRED); **(B)** DV vs. individual prediction (IPRED); **(C)** Conditional weighted residual (CWRES) vs. time; **(D)** CWRES vs. PRED. Red points represent Caucasian population; blue points represent Chinese population; black dashed lines represent reference lines; red solid lines represent loess splines.

The GOF of the final model was evaluated graphically (Figure 4). Overall, scatterplots of observations vs. population or individual predictions indicated that the model adequately described the observations over their entire range. The conditional weighted residuals did not show any obvious trends when plotted against the time or population predictions, indicating that the model was unbiased.

**FIGURE 5:**
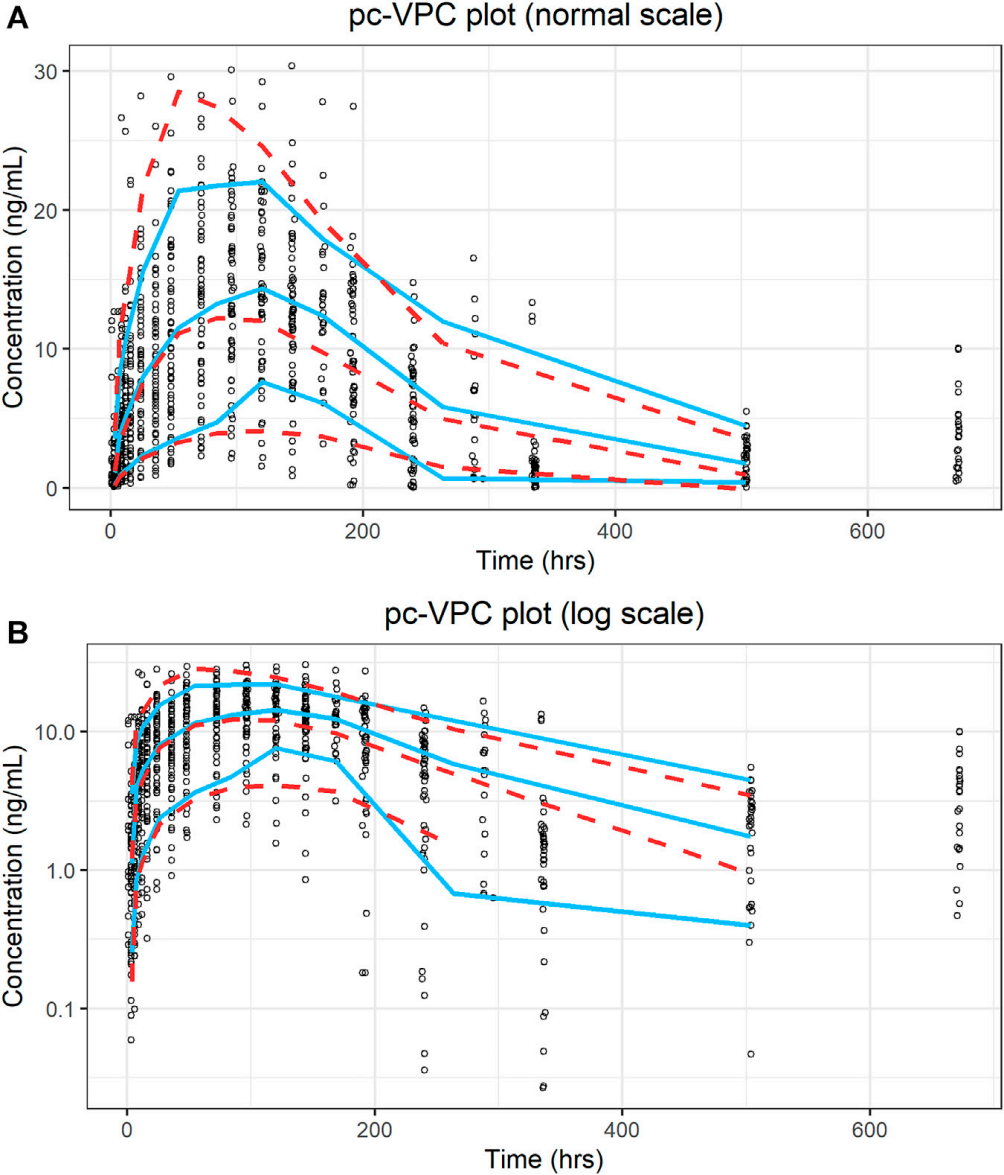
Prediction-corrected visual predictive check plot for the final model. **(A)** Ordinary coordinate; **(B)** Semi-logarithmic coordinate. Blue solid lines represent the 10th, 50th, and 90th percentiles of the observations; red dashed lines represent the 10th, 50th, and 90th percentiles of the simulated values.

A prediction-corrected VPC (pc-VPC) (Bergstrand et al., 2011) was used because of the various dose levels included in this study. The pc-VPC for the final model is shown in Figure 5. In each time period, the 10th, 50th, and 90th percentiles of the observations largely matched the corresponding percentiles of the simulated values.

The non-parametric bootstrapping results are listed in Table 2. The parameter estimates of the final model were within the 90% confidence intervals derived from the bootstrap analysis, and all estimates were close to the bootstrap median. Moreover, the successful estimation rate of the bootstrap was 80%, indicating that the final model was stable.

### Simulation

**FIGURE 6:**
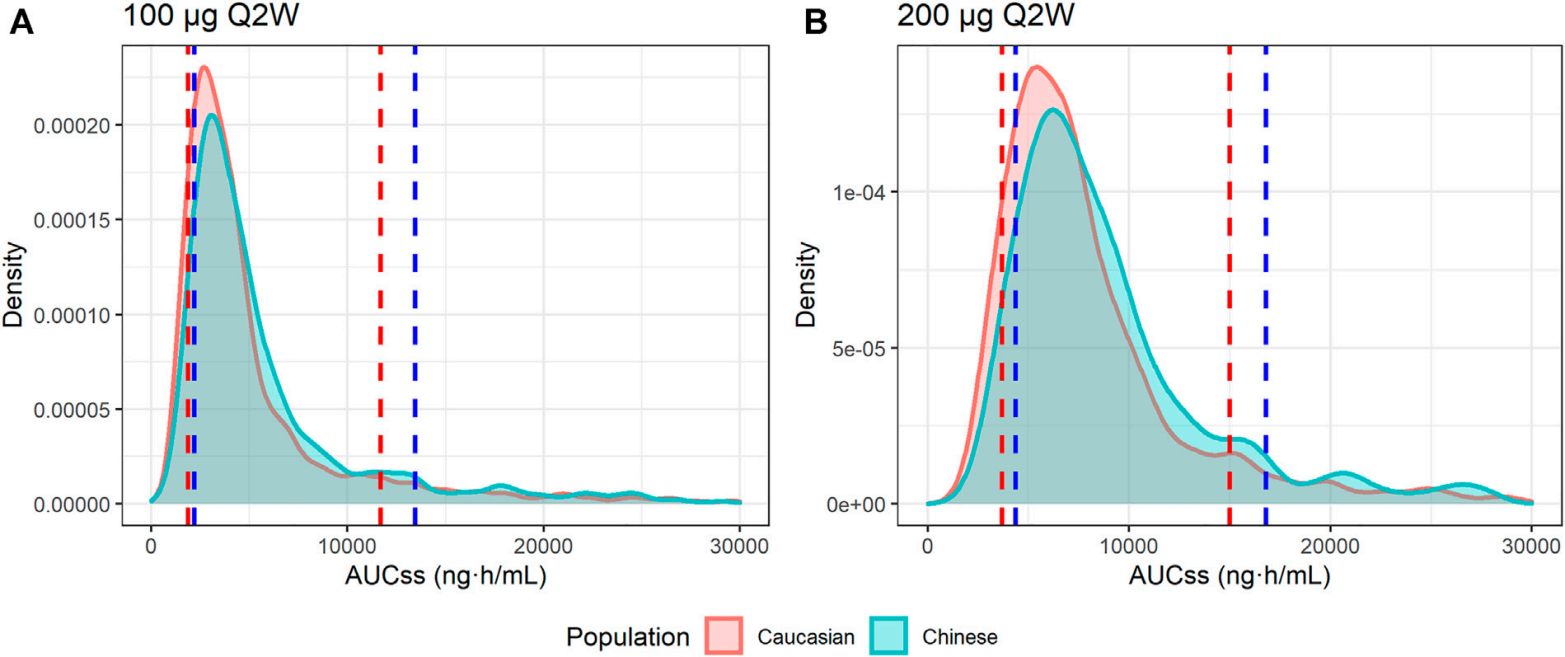
Distributions of the simulated AUC at steady state (AUC_ss_). **(A)** AUC_ss_ of 100 μg every 2 weeks (Q2W); **(B)** AUC_ss_ of 200 μg Q2W. The red area and red dashed lines represent the distribution and 10th and 90th quantiles of the AUC_ss_ of Caucasian virtual subjects, respectively. The blue area and blue dashed lines represent the distribution and 10th and 90th quantiles of AUC_ss_ of Chinese virtual subjects, respectively.

As no obvious PK differences were observed for ropeginterferon alfa-2b between Caucasian and Chinese individuals, and body weight was the only significant factor affecting clearance, two virtual populations were generated by resampling Caucasian and Chinese subjects based on body weight. At 100 μg Q2W, the 80% intervals of the AUC_ss_ were 1,867–11,595 ng h/ml in Caucasian subjects and 2,231–13,403 ng h/ml in Chinese subjects. At 200 μg Q2W, the 80% intervals of the AUC_ss_ were 3,776–14,890 ng h/ml and 4,384–16,922 ng h/ml for Caucasian and Chinese subjects, respectively. As shown in Figure 6, although the AUC_ss_ of the Chinese population was slightly higher than that of the Caucasian population, the distributions of the two groups were very similar. The simulations indicate that a similar exposure could be obtained if Chinese populations were administered the same dose regimen as Caucasians.

## Discussion

We developed the first population PK model of repeginterferon alfa-2b using pooled data to investigate the nonlinear PKs and quantify the potential ethnic difference between Caucasians and Chinese populations. The TMDD model was employed because it has a more mechanistic focus and has better fitness than the empirical MM model. As shown in Eq. 8, ropeginterferon alfa-2b underwent a combination of nonspecific linear elimination ($CL_{linear}$) and receptor-mediated saturable elimination ($CL_{nonlinear}$). The relationship between $C_{free}$ and $K_{D}$ is associated with $CL_{nonlinear}$, leading to a dose-dependent clearance. If the free drug concentration considerably exceeds the value of $K_{D}$, which was estimated at 0.142 ng/ml in this study, the $CL_{nonlinear}$ can be neglected.

Target binding resulting in nonlinear PKs is common for IFNs, and has been reported for INF-β 1a (Mager and Jusko, 2001), IFN-β 1b, and IFN-α 2a (Kagan et al., 2010). However, the TMDD model was not employed to describe the PK of PEG-Intron^®^ (Jen et al., 2001) or Pegasys^®^ (Saleh, 2017) in patients with hepatitis C, which could be attributed to the narrow dose ranges used in these previous studies. The data for model building of PEG-Intron^®^ had three dose levels (0.5, 1, 1.5 μg/kg), and a time-varying clearance model was used. Pegasys^®^ had just one dose (180 μg) and a linear PK elimination model was used. In this study, six dose levels were administrated. The large dose ranges make the dose-dependent PK easily identified.

The median linear *CL/F* was 0.778 L/day for 70-kg subjects, much lower than the reported value of 31.5 L/day in patients with hepatitis C administered PEG-Intron^®^ (Jen et al., 2001), and close to 1.12 L/day in male patients and 0.83 L/day in female patients receiving Pegasys^®^ (Saleh, 2017). The varied clearances of these three long-acting IFNs might be due to differences in their PEGylated moieties. The PEGylated moieties of both ropeginterferon alfa-2b and Pegasys^®^ are approximately 40 kDa, whereas that of PEG-Intron^®^ is 12 kDa. The BSV of ropeginterferon alfa-2b was 35.7%, compared with 40 and 45.9% for PEG-Intron^®^ and Pegasys^®^, respectively. Since all subjects in this study were healthy volunteers, further investigations need to be conducted in the patients.

Body weight was the only significant covariate affecting the PK of ropeginterferon alfa-2b in this study, and no remarkable ethnic differences were observed after adjusting for body weight. The clearance of ropeginterferon alfa-2b increased nonlinearly with body weight and was modeled using a power function with an exponential term of 0.927. The effect of body weight on *CL/F* was consistent with that of PEG-Intron^®^ (Jen et al., 2001). The effect of body weight on the PK of Pegasys^®^ has not been identified (Saleh, 2017).

Sex-based differences in *CL/F* were reported for Pegasys^®^, with higher *CL/F* in males than in females (Saleh, 2017); however, the reason is unknown. The hemoglobin level is a significant factor in the volume of distribution of Pegasys^®^ (Saleh, 2017). However, an effect of hemoglobin levels on the volume of distribution could not be identified in this study, because all subjects were healthy volunteers with normal hemoglobin levels.

This study has a few limitations. Our data were obtained from two phase I clinical trials conducted in healthy subjects, which limits their extrapolation to other populations. In addition, only male Caucasian subjects were enrolled and the effect of sex was not fully investigated. Further investigations in larger populations, especially in patients, are warranted to confirm our results.

## Conclusion

In this study, a population PK analysis was used to quantitatively compare the PKs of ropeginterferon alfa-2b between healthy Chinese and Caucasian subjects. No significant differences in the PK characteristics of ropeginterferon alfa-2b were observed between these populations. The simulation results showed that Chinese are expected to be associated with similar exposures as Caucasians under the same dose.

## Data Availability

The original contributions presented in the study are included in the article/Supplementary Material, further inquiries can be directed to the corresponding author.
