# Supplementary material for: Population Pharmacokinetics of Ropeginterferon Alfa-2b: A Comparison Between Healthy Caucasian and Chinese Subjects
Source: Front Pharmacol. 2021 May 28;12:673492. doi: 10.3389/fphar.2021.673492 (PMC8193675; doi:10.3389/fphar.2021.673492)
Supplement: Supplementary file 1 [file DataSheet1.docx]

Supplementary Material

# Supplementary Tables

Supplementary Table 1. Pharmacokinetic parameters acquired from non-compartment analysis.

| **Parameters** | **Caucasians** | **Chinese** |
| --- | --- | --- |
| **24 μg** |  |  |
| AUC_0–inf_ (ng·h/mL) | 373.97±122.16 | / |
| AUC_0–t_ (ng·h/mL) | 298.12±115.06 |  |
| C_max_ (ng/mL) | 1.82±0.54 |  |
| **48 μg** |  |  |
| AUC_0–inf_ (ng·h/mL) | 620.61±114.58 | / |
| AUC_0–t_ (ng·h/mL) | 391.68±270.61 |  |
| C_max_ (ng/mL) | 2.42±1.42 |  |
| **90 μg** |  |  |
| AUC_0–inf_ (ng·h/mL) | 1243.54±1077.37 | 1280.31±697.12 |
| AUC_0–t_ (ng·h/mL) | 1147.5±1023.89 | 1057.5±721.59 |
| C_max_ (ng/mL) | 5.27±3.24 | 4.63±2.27 |
| **180 μg** |  |  |
| AUC_0–inf_ (ng·h/mL) | 3891.48±976.6 | 3516.24±2254.87 |
| AUC_0–t_ (ng·h/mL) | 3718.03±1075.6 | 3422.24±2158.32 |
| C_max_ (ng/mL) | 20.68±7.66 | 14.63±6.13 |
| **225 μg** |  |  |
| AUC_0–inf_ (ng·h/mL) | 4352.23±1916.5 | / |
| AUC_0–t_ (ng·h/mL) | 4210.42±1942.01 |  |
| C_max_ (ng/mL) | 21.26±8.23 |  |
| **270 μg** |  |  |
| AUC_0–inf_ (ng·h/mL) | 6184.44±1503.67 | 7998.29±2627.03 |
| AUC_0–t_ (ng·h/mL) | 5995.99±1430.53 | 6983.06±2943.77 |
| C_max_ (ng/mL) | 25.21±8.36 | 24.14±8.5 |

AUC_0–inf_: the area under the concentration-time curve from time zero extrapolated to infinity; AUC_0–t_: the area under the concentration-time curve from time zero to the time of the last quantifiable concentration; C_max_: the peak concentration.

Supplementary Table 2. *CL/F*, *V/F*, and *k_a_* estimations with different fixed *k_int_* and *K_D_* values

|  | **Original** | **0.5 times** | **0.75 times** | **1.25 times** | **2 times** |
| --- | --- | --- | --- | --- | --- |
| **k_int_ fixed values (h^-1^)** | **0.0788** | **0.0394** | **0.0591** | **0.118** | **0.158** |
| CL/F estimates (L/day) | 0.778 | 0.843 | 0.8 | 0.794 | 0.748 |
| Changes (%) | / | 8.2 | 2.8 | 2.1 | -3.9 |
| V/F estimates (L) | 2.32 | 2.61 | 2.4 | 1.94 | 2.2 |
| Changes (%) | / | 12.5 | 3.4 | -16.4 | -5.2 |
| k_a_ estimates (day^-1^) | 0.14 | 0.151 | 0.143 | 0.128 | 0.136 |
| Changes (%) | / | 7.9 | 2.1 | -8.6 | -2.9 |
| **K_D_ fixed values (ng/mL)** | **0.142** | **0.71** | **0.106** | **0.213** | **0.284** |
| CL/F estimates (L/day) | 0.778 | 0.815 | 0.777 | 0.779 | 0.78 |
| Changes (%) | / | 4.8 | -0.1 | 0.1 | 0.3 |
| V/F estimates (L) | 2.32 | 1.92 | 2.33 | 2.31 | 2.29 |
| Changes (%) | / | -17.2 | 0.4 | -0.4 | -1.3 |
| k_a_ estimates (day^-1^) | 0.14 | 0.131 | 0.14 | 0.14 | 0.139 |
| Changes (%) | / | -6.4 | 0 | 0 | -0.7 |

*CL/F*: apparent clearance; *V/F*: apparent volume of distribution; *k_a_*: absorption rate; *k_int_*: internalisation rate of drug-recepter complex; *K_D_*: equilibrium dissociation constant of drug-receptor binding.

Supplementary Table 3. Stepwise covariates selection.

| **Covariates** | **Parameters** | **OFV** | **Base OFV** | **dOFV** | ***df*** | ***p*-value** | **Significant** |
| --- | --- | --- | --- | --- | --- | --- | --- |
| **Forward inclusion step 1** | | | | | | | |
| Ethnicity | CL/F | 1552.198 | 1553.69 | -1.492 | 1 | 0.222 |  |
| Sex | CL/F | 1550.811 | 1553.69 | -2.879 | 1 | 0.09 |  |
| Weight | CL/F | 1454.381 | 1553.69 | -8.309 | 1 | 0.004 | Yes |
| Ethnicity | k_a_ | 1549.364 | 1553.69 | -4.326 | 1 | 0.038 | Yes |
| Sex | k_a_ | 1548.753 | 1553.69 | -4.937 | 1 | 0.026 | Yes |
| **Forward inclusion step 2** | | | | | | | |
| Ethnicity | CL/F | 1545.224 | 1454.381 | -0.157 | 1 | 0.692 |  |
| Sex | CL/F | 1544.783 | 1454.381 | -0.598 | 1 | 0.439 |  |
| Ethnicity | k_a_ | 1543.847 | 1454.381 | -1.534 | 1 | 0.216 |  |
| Sex | k_a_ | 1542.922 | 1454.381 | -2.459 | 1 | 0.117 |  |
| **Backward elimination step 1** | | | | | | | |
| Weight | CL/F | 1448.934 | 1540.302 | +8.309 | 1 | 0.004 | Yes |

OFV: objective function value; *df*: degree of freedom; *CL/F*: apparent clearance; *k_a_*: absorption rate.
